# Commentary: Preconditioning tDCS facilitates subsequent tDCS effect on skill acquisition in older adults

**DOI:** 10.3389/fnagi.2017.00084

**Published:** 2017-03-31

**Authors:** George M. Opie, John Cirillo

**Affiliations:** ^1^Discipline of Physiology, Adelaide Medical School, The University of AdelaideAdelaide, SA, Australia; ^2^Movement Neuroscience Laboratory, Department of Exercise Sciences, The University of AucklandAuckland, New Zealand; ^3^Centre for Brain Research, The University of AucklandAuckland, New Zealand

**Keywords:** metaplasticity, aging, TMS/tDCS, motor training, neuroplasticity

Long-term potentiation and depression (LTP and LTD) represent powerful means of altering synaptic communication. However, they have an inherent potential to excessively modulate neuronal excitability, possibly resulting in unfavorable destabilization of neural function. It has been suggested that homeostatic regulation (i.e., metaplasticity) of neuronal networks avoids such destabilization. This construct proposes that the threshold for modification of neuronal excitability is based on previous post-synaptic activity, with an elevated threshold following high neuronal activity, but a reduced threshold following low neuronal activity (Bienenstock et al., 1982). Studies using non-invasive brain stimulation (NIBS) over human primary motor cortex (M1) support the utilization of such a mechanism (Karabanov et al., 2015). Furthermore, NIBS plasticity interventions have demonstrated an ability to interact homeostatically with motor learning, a process that is at least partially LTP-dependent (Ziemann et al., 2004; Jung and Ziemann, 2009). This has led to the exciting possibility of using “priming” NIBS to enhance the response to motor training following injury, or compensate for deficits in neuroplastic capacity. While relevant to many different populations, such an approach is of particular interest in older adults, where deficits in neuroplastic capacity (Barnes, 2003) and motor learning (Voelcker-Rehage et al., 2006) are well defined. However, it is currently unknown whether priming stimulation remains effective for potentiating the response to training in the elderly.

In a recent paper within *Neurobiology of Aging*, Fujiyama and colleagues (Fujiyama et al., 2017) attempted to address this gap in knowledge by utilizing transcranial direct current stimulation (tDCS; a NIBS technique able to reliably induce LTP- and LTD-like plasticity in M1) to prime skill acquisition in young and older adults. The study utilized a visuomotor training task that involved moving a cursor on a computer screen through a range of targets by abducting the non-dominant index finger against a force transducer. Each target required a different level of force and target order was consistent across trials. During training, all subjects received 20 mins of excitatory anodal tDCS (atDCS). However, prior to training, half received 10 min of inhibitory cathodal tDCS (ctDCS), whereas the other half received 10 min of sham stimulation. Single- and paired-pulse TMS over the right M1 was used to quantify corticomotor excitability (motor evoked potential (MEP) area), and intracortical function (short-interval intracortical inhibition, SICI; intracortical facilitation, ICF), respectively. Changes in neurophysiological measures and motor performance were assessed as outcome variables. The results indicated that both young and older adults improved in performance following training, with a greater magnitude of effect observed in young subjects. Furthermore, performance following priming ctDCS was greater than sham in both groups. In addition, motor training potentiated corticospinal excitability in both groups, but only when preceded by ctDCS. In contrast, there was no modulation of ICF or SICI following motor training. The authors suggest that these findings indicate a maintenance of metaplastic mechanisms in the elderly, while also demonstrating the utility of priming stimulation for improving the response to training in older adults.

While Fujiyama and colleagues are the first to investigate whether aging impairs the capacity of priming stimulation to modulate the response to motor training, another recent study used paired blocks of theta burst stimulation (TBS; a TMS paradigm able to induce LTP- and LTD-like changes in corticospinal excitability) to investigate age-related changes in the mechanisms of metaplasticity (Opie et al., 2017). In contrast to Fujiyama et al., this study reported that priming was ineffective in older adults. The cause of these discrepant findings is currently unclear. However, differences in the effectiveness of the applied NIBS techniques for modulating excitability in M1 may be a factor, possibly suggesting a greater clinical utility of tDCS for the metaplastic modulation of skill acquisition in older adults. One factor contributing to this outcome may be that tDCS was applied during task performance, whereas TBS was applied at rest. Subsequently, tDCS was given at a time when neural activation of task-related muscles was increased to successfully execute the movement, which likely influenced the plasticity response (Ridding and Ziemann, 2010). In addition, the conventional tDCS montage used by Fujiyama et al. is less focal (i.e., stimulates a larger cortical area) than other NIBS techniques (such as TBS), and may have resulted in activation of cortical networks other than M1 that could be important in motor learning. Therefore, the metaplastic modulation of corticospinal excitability observed by Fujiyama and colleagues may have stemmed from their utilized intervention producing a stronger and more widespread activation of motor cortical networks. The conflicting findings between these studies may therefore indicate that the aging process results in an increased threshold for inducing metaplastic effects.

While the cause of these conflicting findings may be interesting, the real challenge of priming interventions is ensuring that whether motor improvements observed within the lab can be functionally translated to everyday life. This question highlights the volume of work that remains to fully elucidate the role that priming stimulation may have in the field of aging. There are several parameters that require investigation to help answer this question. For example, can motor improvement in older adults be made stronger, more stable or longer lasting by applying additional blocks of priming stimulation? How critical is the lapsed time between priming and training? Are alternative approaches that change neuronal excitability immediately prior to modulating synaptic strength (a notion referred to as “gating” that reflects a non-homeostatic effect of excitability-enhancement) (Ziemann and Siebner, 2008) more effective for potentiating the plasticity response? Can homeostatic and non-homeostatic mechanisms be combined with metaplastic principles to produce an optimal outcome? Despite the current uncertainty surrounding priming interventions and their functional relevance with advancing age, the findings of Fujiyama and colleagues demonstrate that it is possible to metaplastically potentiate the response to motor training in older adults. While the findings should be considered preliminary until repeated within a larger cohort using a between-subjects design, they represent an interesting and exciting contribution to the field. Ultimately, identifying metaplastic processes optimal for motor learning in older adults may offer novel insights about the use of priming approaches for neurorehabilitation.

## Author contributions

GO and JC both conceptualized, drafted, revised, and approved manuscript.

### Conflict of interest statement

The authors declare that the research was conducted in the absence of any commercial or financial relationships that could be construed as a potential conflict of interest. The reviewer AR and handling Editor declared their shared affiliation, and the handling Editor states that the process nevertheless met the standards of a fair and objective review.
